# Description of an Acephalous Fœtus

**Published:** 1827-02

**Authors:** Robert Abraham

**Affiliations:** Surgeon, Carlisle.


### ACEPHALOUS FŒTUS.

*Description of an Acephalous Fœtus.* By Robert Abraham, Surgeon, Carlisle.

On the 2d of December, 1826, Mrs. B—, of this place, was delivered of a fœtus of the presumed age of seven months. When it was born, it showed not the smallest signs of life, excepting an obscure pulsation in the umbilical cord, which ceased immediately on its birth.

As I believed, from its external appearance, that an accurate examination of it would prove extremely interesting, I obtained permission to open it; which I did with the assistance of Dr. Barnes of this place, whose intimate knowledge of the cerebral structures, and skill in developing them, I have had frequent opportunities of observing.

The limbs and body were those of a fully grown fœtus of the age of seven or eight months, but the upper part of the head was entirely deficient; the whole of the parietal bones, and nearly all the squamous portion of the temporal, being wanting; and also all that part of the front of the cranium superior to the eyes, and all the occipital bone, excepting the basilar and condyloid processes; the spinous and transverse processes of the four superior cervical vertebræ were also wanting, so that the spinal canal was laid completely open.

The eyes were covered with palpebræ, and, from the deficiency of the frontal bone, formed the superior boundary of the face: they stood wide open, and, from their size and position, gave the countenance a very hideous aspect. There was a little hair on the temple.

The back part of the head was covered by a delicate membrane, extending over the deficiency in the spine. It was of a dark mulberry colour, and was gorged with blood, as were likewise the eyes; so that the labour had probably propelled more blood into those parts than was natural to them. On dividing this membrane, it was found to cover a large venous plexus with considerable sinuses, so that in a short time all the fluids in the body were evacuated through them. Beneath them lay a dura mater, and the basilar spheno-occipital process very completely ossified; but the most rigid examination could not detect even the rudiments of a brain. The optic nerves terminated without uniting immediately after leaving the sclerotica; the sphenal foramina were imperforate, the body of the bone bearing a pretty accurate resemblance to the ordinary shape of the posterior part of the atlas; the petrous portion of the temporal bone was incomplete, though the external ear was perfect; the nerves of the neck were seen losing themselves beneath the base of the skull; the spinal cord terminated at the cervical vertebræ. *Nothing like a cerebrum, cerebellum, or medulla oblongata, was discernible.*

The parents are well formed; this was the fourth pregnancy. Two of the children are dead: the survivor, a boy, is a beautiful child, with a finely developed cranium. The labour, in its first stage, was tedious and difficult; remarkable, in an extreme degree, for irregular dilatation and rigidity of the os uteri. It exceeded any I ever before witnessed in tension of the membranes and copiousness of liquor amnii. The umbilical cord was very short, and the placenta small.

I am not fond of mixing facts and controversy; yet I trust my readers will excuse my observing, that this case furnishes no confirmation of the theory lately advanced by M. Geoffroy St. Hilaire. There was not the slightest vestige of a twin; and, from circumstances unnecessary to detail, I am satisfied I could not be mistaken on that point.

*Carlisle; December 15th,* 1826.

